# Case Report: Lenvatinib-induced lichenoid drug reaction mimicking disseminated zoster in a patient with metastatic hepatocellular carcinoma

**DOI:** 10.3389/fonc.2026.1905649

**Published:** 2026-09-16

**Authors:** Maria Kaufman, Rodrigo Garcia-Santisteban, Crystal Choi, Rohit Merugu, Samuel Kareff

**Affiliations:** 1 Miller School of Medicine, University of Miami, Miami, FL, United States; 2 Department of Internal Medicine, School of Medicine, Florida Atlantic University, Boca Raton, FL, United States; 3 Lynn Cancer Institute, Baptist Health South Florida, Boca Raton, FL, United States

**Keywords:** case report, cutaneous adverse events, hepatocellular carcinoma, lenvatinib, lichenoid drug reaction, tyrosine kinase inhibitor

## Abstract

Lenvatinib, a multi-kinase inhibitor targeting VEGFR 1–3 and FGFR 1–4, is a first-line therapy for unresectable hepatocellular carcinoma (HCC). While palmar-plantar erythrodysesthesia and generalized erythematous rashes are recognized side effects, lenvatinib-induced lichenoid drug reactions (LDRs) remain infrequently reported and pose significant diagnostic challenges when presenting as viral exanthematous mimics. This case describes an 82-year-old female with metastatic HCC who developed a generalized, pruritic, and vesicular rash mimicking disseminated herpes zoster shortly after initiating lenvatinib. Despite prior shingles vaccination, empiric intravenous acyclovir was initiated. A skin biopsy revealed a lichenoid dermatitis with Civatte bodies and significant eosinophilia, confirming an LDR. The eruption resolved following systemic corticosteroid therapy, indicating a likely reaction to recent lenvatinib exposure. This case highlights the importance of histopathological verification and chronological drug reconciliation in differentiating drug-induced dermatotoxicity from viral exanthems in fragile oncology populations.

## Introduction

The therapeutic landscape for unresectable or metastatic hepatocellular carcinoma (HCC) has shifted toward the use of immune checkpoint inhibitors (ICIs) and multi-kinase inhibitors, which have significantly improved survival outcomes (1–4). Lenvatinib, an oral tyrosine kinase inhibitor (TKI) targeting vascular endothelial growth factor receptors (VEGFRs) 1–3 and fibroblast growth factor receptors (FGFRs) 1–4, is an NCCN-preferred first-line option (1, 3). While effective, lenvatinib is associated with adverse events, including hypertension, gastrointestinal symptoms, fatigue, and cutaneous toxicities (5, 6).

Cutaneous adverse events (cAEs) from targeted oncological therapies often manifest as palmar-plantar erythrodysesthesia (PPE), generalized erythematous rash (GER), or alopecia (7–9). Lichenoid drug reactions (LDRs), characterized by symmetric, erythematous, or violaceous papules developing after a latency period (10, 11), are well-documented with ICIs and certain TKIs (2, 10). Across the ICI class, LDRs represent one of the most common immune-related cAEs, reported in up to 17% of patients, though this is primarily driven by anti-PD-1 agents rather than anti-PD-L1 antibodies (e.g., atezolizumab) (12, 13). Concurrently, while TKIs represent the second most frequent class implicated in lichenoid tissue reactions after checkpoint inhibitors (10), cutaneous lichenoid eruptions specifically attributed to lenvatinib remain infrequently reported in the oncology literature (14–16).

Here, we describe a generalized lichenoid eruption in an 82-year-old female following sequential atezolizumab and lenvatinib therapy, highlighting the role of histopathological verification and chronological drug reconciliation to differentiate between drug-induced dermatotoxicity and viral exanthems, such as varicella-zoster virus (VZV), in the immunocompromised oncology population.

## Case description

An 82-year-old female with stage 4 chronic kidney disease (CKD) secondary to polycystic kidney disease, cirrhosis secondary to hepatitis C, Crohn’s disease status post total colectomy, and metastatic poorly differentiated HCC presented to the emergency department on 31 March 2026 for evaluation of a generalized, pruritic rash.

After being diagnosed with HCC in April 2022, she was managed sequentially with partial hepatic lobectomy, Y-90 radioembolization, and stereotactic body radiotherapy (**Table 1**). Following progression at the gastroesophageal junction in October 2025, she received three cycles of first-line atezolizumab (1200 mg) and bevacizumab (868 mg) from 24 November 2025 to 12 January 2026. Due to disease progression, second-line oral lenvatinib was initiated on 18 February 2026 at a weight-adjusted dose of 8 mg daily. Lenvatinib was administered from 18 February to 7 March 2026, briefly held due to hospitalization, resumed at 4 mg daily from 10 March to 18 March 2026, and then held indefinitely due to grade 3 fatigue and an acute-on-chronic kidney injury.

**Table 1:** Chronological timeline of oncology treatments, diagnostic findings, and clinical interventions.

Date	Clinical presentation and diagnostics	Intervention and outcome
April 2022	Initial hepatic mass presentation	Partial hepatic lobectomy; poorly differentiated HCC confirmed
June 2022	Recurrent viable liver tumor on MRI	Y-90 radiation therapy with localized disease control
November 2024	Gastrohepatic mass (3.4 cm × 2.1 cm) on upper endoscopy	SBRT with localized disease control
October 2025	Infiltrative 30 mm gastroesophageal mass on upper endoscopy	Biopsy confirmed metastatic HCC
24 November 2025–12 January 2026	First-line systemic immunotherapy regimen	Atezolizumab (1,200 mg) and bevacizumab (868 mg); disease progression by February 2026
18 February–7 March 2026	Second-line targeted oral TKI regimen	Lenvatinib 8 mg daily; held briefly due to hospitalization
10 March–18 March 2026	Dose-reduced oral TKI continuation	Lenvatinib 4 mg daily; held indefinitely due to grade 3 fatigue and AKI
Mid-late March 2026	Cutaneous eruption onset: generalized, pruritic rash without mucosal involvement (~1–2 weeks post-lenvatinib cessation; ~10 weeks post-atezolizumab)	–
31 March 2026 (HD 1)	ED presentation: blanchable, erythematous rash with healing vesicles and dry, flaky skin	Empiric IV acyclovir and diphenhydramine for suspected disseminated VZV
2 April 2026 (HD 3)	Right shoulder punch biopsy performed due to lack of response to antiviral therapy	Acyclovir continued pending pathology
6 April 2026 (HD 7)	Surgical pathology demonstrated lichenoid interface infiltrate, basal vacuolization, necrotic keratinocytes (Civatte bodies), and significant eosinophilia, supporting an LDR	IV acyclovir discontinued; 6-day oral methylprednisolone taper initiated; rash improved
Discharge and follow-up	Planned oncology treatment holiday followed by potential lenvatinib rechallenge at 4 mg daily	Rechallenge never attempted due to functional decline; transitioned to hospice care in May 2026

HCC, hepatocellular carcinoma; MRI, magnetic resonance imaging; SBRT, stereotactic body radiation therapy; TKI, tyrosine kinase inhibitor; AKI, acute kidney injury; HD, hospital day; ED, emergency department; IV, intravenous; VZV, varicella-zoster virus, LDR, lichenoid drug reaction.

The cutaneous eruption began in mid-to-late March 2026, approximately 1–2 weeks following lenvatinib discontinuation (4–5 weeks after initiation). The rash was diffusely distributed across the upper chest, upper back, upper extremities, and anterior thighs without mucosal involvement (**Figure 1**). The rash was associated with intense pruritus, fatigue, and generalized weakness. The patient denied fevers, chills, chest pain, headache, shortness of breath, nausea, or vomiting. Physical exam revealed a blanchable, erythematous rash with evidence of healing vesicles and areas of dry, flaky skin in various stages of ecchymosis. Admission labs revealed mild leukocytosis (11.5 K/µL), mild thrombocytopenia (115 K/µL), and a moderate pre-renal acute kidney injury with a serum creatinine of 3.60 mg/dL (baseline 2.3 mg/dL–2.8 mg/dL).

**Figure 1:**
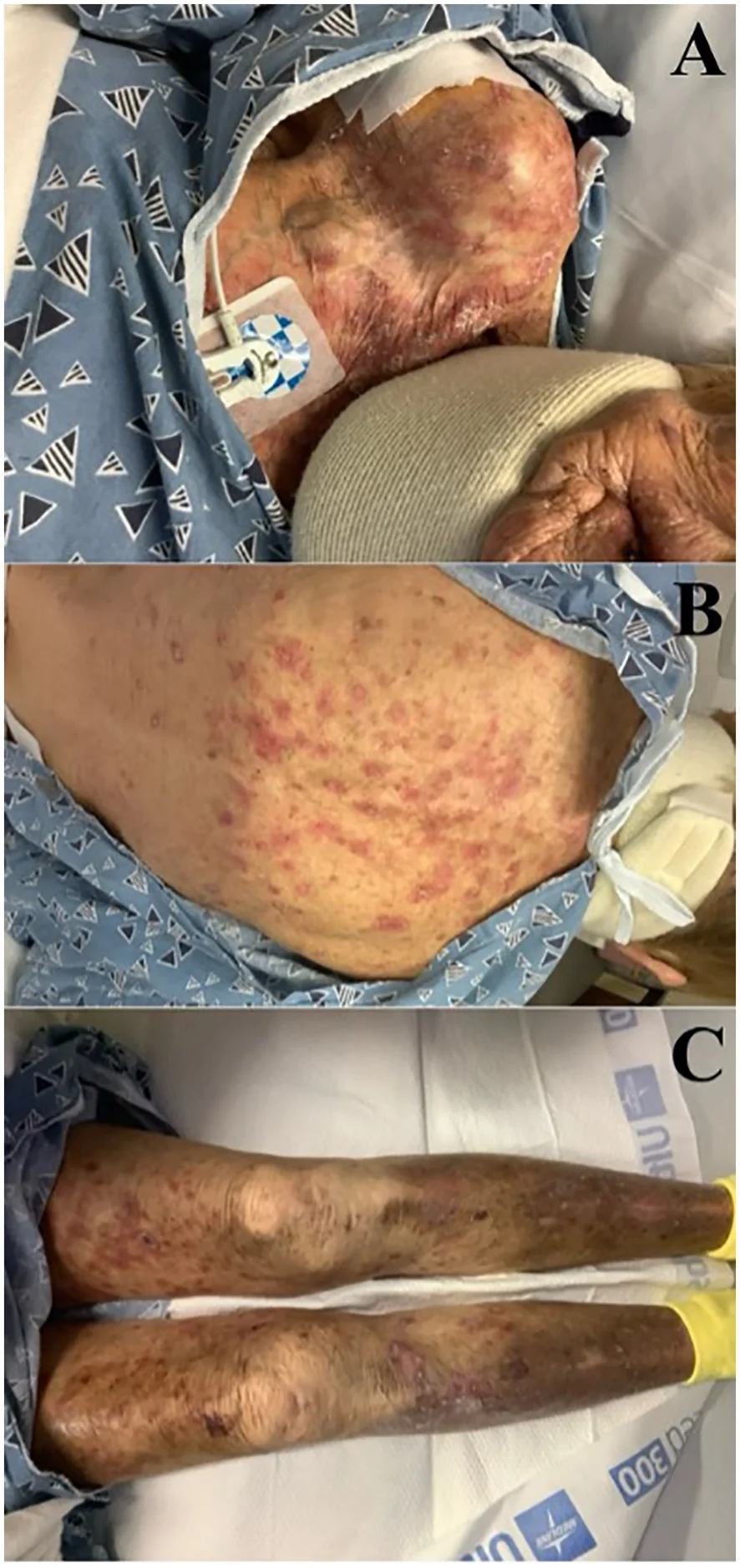
Close-up clinical photograph of the upper chest and right shoulder **(A)**, posterior torso **(B)**, and bilateral lower extremities **(C)** demonstrating a diffuse, pruritic lichenoid exanthem with dry, flaky skin, scattered hyperpigmented macules, and focal areas of resolving inflammation.

Initially, the presentation raised concern for a disseminated VZV infection, despite prior vaccination. Given the patient’s oncologic exposures, drug-induced etiology, erythema multiforme, an autoimmune reaction, and paraneoplastic syndrome were also considered. The patient was empirically started on intravenous acyclovir and diphenhydramine. No dedicated viral swab PCR, Tzanck smear, or viral cultures were obtained based on recommendations from an infectious disease consultant. Confounding medications included a recently completed course of ciprofloxacin for a urinary tract infection (started after rash onset), distant atezolizumab exposure, and long-standing regimens of levothyroxine, allopurinol, rosuvastatin, and pantoprazole.

Due to the lack of clinical improvement on acyclovir, a bedside skin biopsy of the right shoulder was performed on hospital day 3. Surgical pathology (H&E) revealed interface dermatitis with basal keratinocyte destruction, apoptotic/necrotic Civatte bodies, and a lichenoid inflammatory infiltrate with significant eosinophilia (**Figure 2**), findings diagnostic of a lichenoid drug reaction. No viral cytopathic effects or subepidermal bullae were identified. Acyclovir was discontinued, and a 6-day course of oral methylprednisolone was initiated (24 mg on day 1, tapered by 4 mg daily). Renal function improved to baseline with fluid resuscitation, and the rash began to resolve. The patient was discharged with plans for a treatment holiday followed by reinitiation of lenvatinib at 4 mg daily. However, lenvatinib was never rechallenged due to subsequent functional decline, and she was transitioned to hospice care in May 2026 (**Table 1**).

**Figure 2:**
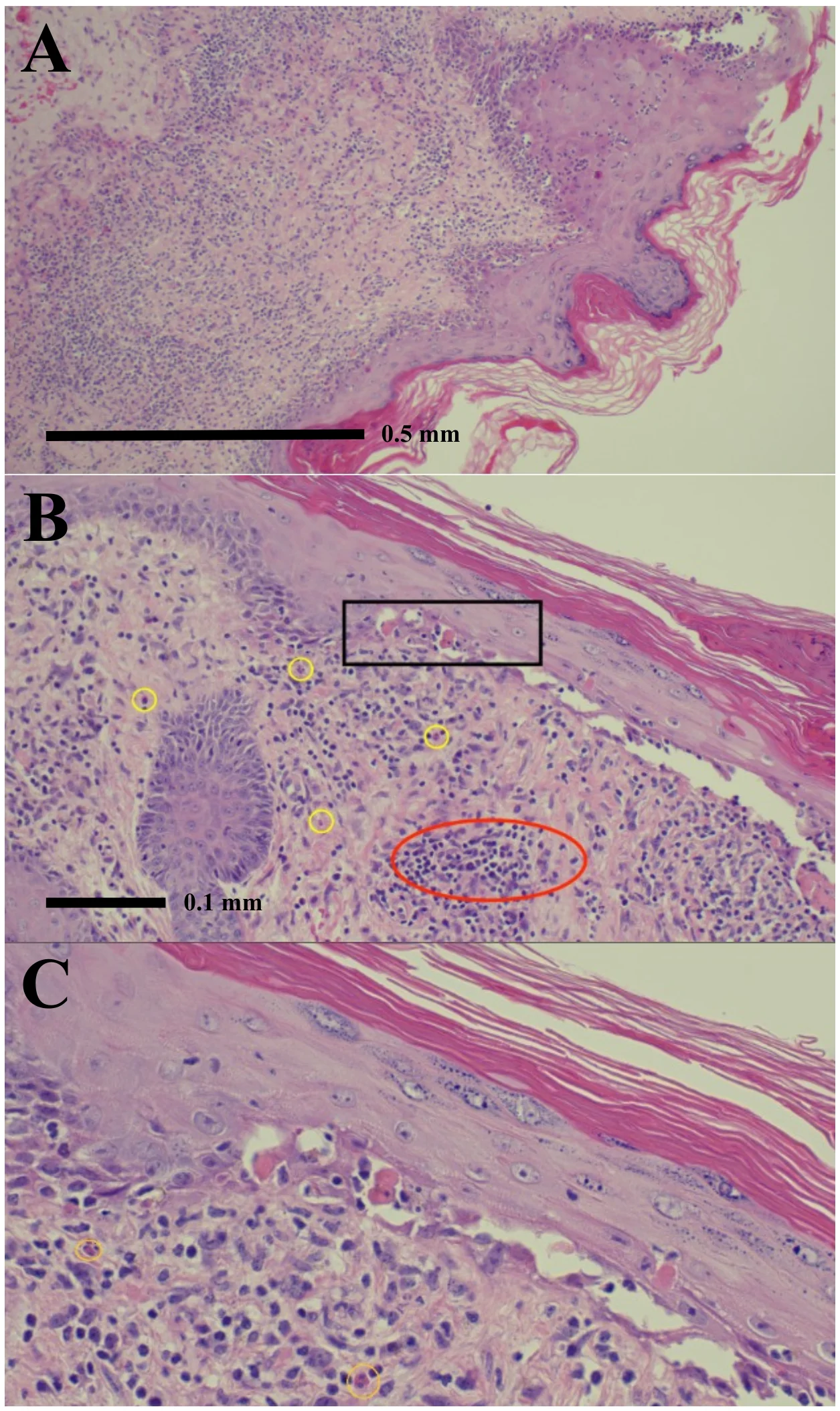
Histopathological findings of the skin punch biopsy (H&E stain). **(A)** Low-power view (×100 magnification, scale bar = 0.5 mm) demonstrating hyperkeratosis, hypogranulosis, and a dense, band-like lichenoid inflammatory infiltrate in the upper dermis. **(B)** Medium-power view (×200 magnification, scale bar = 0.1 mm) showing perivascular lymphocytic infiltration (red circle), significant eosinophilia (yellow circles), and interface dermatitis with basal cell vacuolar degeneration and focal dyskeratosis (black box). **(C)** High-power view (×400 magnification) demonstrating necrotic and apoptotic keratinocytes (Civatte bodies) along the dermoepidermal junction, alongside infiltrating lymphocytes and significant eosinophilia (yellow circles).

## Discussion

Generalized, vesicular rash in elderly immunocompromised cancer patients necessitates the immediate exclusion of disseminated VZV. In this case, prior vaccination did not entirely eliminate suspicion, prompting empiric antiviral therapy. However, the lack of response to acyclovir and subsequent histopathological examination supported a drug-induced etiology. Nonetheless, because dedicated viral PCR or Tzanck testing was not obtained, an atypical viral presentation could not be unequivocally ruled out on histology alone. Distinguishing between a primary viral exanthem and a drug-induced eruption is critical as systemic corticosteroids, the cornerstone of treatment for severe drug reactions (8, 9), can precipitate life-threatening viral dissemination if administered during active VZV infection (9, 10).

cAEs associated with TKIs like lenvatinib arise primarily from the inhibition of VEGFR and FGFR signaling pathways essential for epidermal and vascular homeostasis (7, 17). Although PPE and GER are most common, more severe and rare manifestations, including pemphigus erythematosus, ulcerative skin lesions, psoriasiform reaction, acral skin peeling syndrome, and pyoderma gangrenosum, have been documented (14, 15, 18–25). Biopsy findings of a lichenoid interface dermatitis with basal keratinocyte vacuolization, Civatte bodies, and prominent dermal eosinophilia are characteristic of a lichenoid tissue reaction (10).

Determining the precise culprit between atezolizumab and lenvatinib was the principal diagnostic challenge. While the patient had also received bevacizumab, anti-VEGF monoclonal antibodies are not typically associated with lichenoid tissue reactions (26). Furthermore, while long-standing maintenance medications (allopurinol, rosuvastatin, and pantoprazole) can induce LDRs, their chronic use prior to presentation makes acute sensitization less probable. Retrospective causality assessment yielded a Naranjo probability score of 4 and a “possible association” under WHO-UMC criteria for both antineoplastic agents.

Because both ICIs and TKIs can generate histologically similar LDRs, chronological drug reconciliation is essential. ICI-induced cutaneous toxicities frequently manifest within 2–6 weeks of starting therapy (26); however, delayed immune-related adverse events (irAEs) can develop or persist months after drug cessation due to sustained T-cell activation against dermo-epidermal antigens (2). Specifically, ICI-associated lichenoid eruptions exhibit a highly variable latency, occasionally arising weeks to months following drug discontinuation (27–29). In contrast, TKI-associated dermatotoxicity follows a more acute and predictable timeline, typically developing within 2–4 weeks of drug initiation (30–32) and coinciding with peak steady-state drug exposure. In this patient, the rash developed approximately 4–5 weeks after initiating lenvatinib and 1–2 weeks after its withdrawal, compared to an approximately 10-week latency following the last atezolizumab infusion. While late-onset atezolizumab toxicity cannot be entirely excluded, the acute clinical timeline aligns more closely with the typical onset window of oral TKI-induced dermatotoxicity. Thus, we favor lenvatinib as the more probable, albeit nondefinitive, acute trigger and note that the co-administration of both agents may also have led to the patient’s presentation.

Management of targeted therapy-induced LDRs typically involves topical or systemic corticosteroids and discontinuation or dose reduction of the offending drug (8, 9). In this patient, the severity of the rash prompted oral methylprednisolone use, leading to clinical improvement. When resuming therapy, dose reduction or intermittent dosing schedules (“weekends off”) for lenvatinib can improve tolerability without compromising therapeutic response in HCC (33). A multidisciplinary approach involving oncology, infectious disease, and cutaneous surgery is vital for rapid diagnosis and appropriate therapeutic guidance. As targeted therapies become more prevalent, clinicians must remain vigilant for atypical cutaneous presentations like LDR to ensure timely intervention and avoid unnecessary morbidity in oncologic patients.

Several limitations warrant consideration. The absence of a formal drug rechallenge due to performance status decline and the presence of confounding polypharmacy from sequential atezolizumab and chronic medications preclude absolute single-agent confirmation. Furthermore, while physical examination, clinical distribution, and histopathology strongly supported a drug reaction, specific viral swab PCR or Tzanck testing was not performed during the acute presentation to definitively exclude a concurrent atypical viral exanthem.

## Data Availability

The original contributions presented in the study are included in the article/supplementary material. Further inquiries can be directed to the corresponding author.
